# The association of primary aldosteronism with glaucoma-related fundus abnormalities

**DOI:** 10.1371/journal.pone.0242090

**Published:** 2020-11-06

**Authors:** Yoshimi Ohshima, Tomomi Higashide, Kimikazu Sakaguchi, Makoto Sasaki, Sachiko Udagawa, Shinji Ohkubo, Takashi Yoneda, Kazuhisa Sugiyama

**Affiliations:** 1 Department of Ophthalmology, Kanazawa University Graduate School of Medical Science, Kanazawa, Japan; 2 Ohkubo Eye Clinic, Kanazawa, Japan; 3 Department of Cardiovascular and Internal Medicine, Kanazawa University Graduate School of Medical Science, Kanazawa, Japan; Bascom Palmer Eye Institute, UNITED STATES

## Abstract

**Purpose:**

To elucidate glaucoma-related fundus abnormalities in patients with primary aldosteronism (PA).

**Methods:**

The study compared 272 eyes from 137 PA patients and 352 eyes from 177 control subjects selected randomly from 1173 participants of a public glaucoma screening. The presence of glaucomatous optic disc appearance (rim thinning and cup enlargement) and retinal nerve fiber layer defects (RNFLDs) was determined independently from fundus photographs. The results were compared between the PA and control groups.

**Results:**

There were 9 patients (6.6%) with glaucomatous optic disc abnormalities in the PA group and 10 cases (5.6%) identified in the control group (p = 0.92). RNFLDs were detected more frequently in the PA group (55 eyes, 20.2%) than in the control group (26 eyes, 7.4%; p<0.001). The two types of RNFLDs were classified as either having their central ends at the disc margin (D) or away from the disc margin and around the retinal vessels (V). Type D and V RNFLDs were detected in 35 (12.9%) and 26 (9.6%) eyes in the PA group and in 25 (7.1%) and 4 (1.1%) eyes in the control group, respectively. Both types of RNFLDs were more frequent in the PA group than in the control group (Type D and V, p = 0.03, <0.001, respectively).

**Conclusion:**

Although the prevalence of glaucomatous optic disc appearance did not differ between the two groups, RNFLDs were more frequent in PA patients than in the control group. Moreover, RNFLDs with their central ends located around retinal vessels were characteristic of PA patients.

## Introduction

Primary aldosteronism (PA) is characterized by the overproduction of aldosterone by the adrenal glands and typically causes hypertension, cardiovascular damage, sodium retention, suppression of plasma renin and hypokalemia [1, 2]. The frequency of PA is about 5% (1.6% - 11.2%) in hypertension, and about 20% (11.3% - 31%) in hypertension with various complications. Patients with PA are at higher risk for cardiovascular morbidity and mortality than patients with essential hypertension accounting for age, gender, and systolic and diastolic blood pressure [1, 3, 4]. The number of patients with hypertension was estimated to be 43 million in Japan based on a national survey conducted in 2010 [5], which equates to approximately 2 million patients with PA currently living in Japan. As PA often causes hypertension, PA patients may be at risk for hypertensive retinopathy and other hypertension-related eye diseases such as retinal vascular occlusion and non-arteritic anterior ischemic optic neuropathy [6, 7]. Furthermore, several case reports have indicated an association between PA and central serous chorioretinopathy [8]. However, a detailed characterization of fundus abnormalities in PA patients and the relationship with glaucoma have yet to be conducted.

Nitta et al. reported that systemic administration of aldosterone to rats resulted in progressive retinal ganglion cell (RGC) loss and glaucomatous optic nerve degeneration without intraocular pressure (IOP) elevation; this raised the possibility that aldosterone may also have a role in RGC death in human normal-tension glaucoma (NTG) [9].

Glaucoma is one of the leading causes of visual loss, and the prevalence of glaucoma in the Japanese population older than 40 years of age was estimated to be 5.0% [10, 11]. Glaucomatous damage specifically affects RGCs and their axons leading to progressive thinning of the retinal nerve fiber layer (RNFL) accompanied by structural changes within the optic nerve head [12]. These changes can be detected as RNFL defects (RNFLDs) and optic disc rim thinning in fundus images [13, 14]. It is well known that increased intraocular pressure (IOP) is one of the most important risk factors for glaucoma; however, the basic pathological processes of glaucoma, especially in NTG, have yet to be fully elucidated. NTG sometimes shows disease progression despite a reduction in IOP [15], and clinical factors other than IOP, such as vascular dysregulation, are suggested to be some of the mechanisms involved in glaucoma progression [16]. Non-IOP-related cardiovascular dysregulation factors, such as systemic hypertension, systemic hypotension, nocturnal hypotension, and cardiac arrhythmia are reported to be risk factors for NTG [17]. PA may also be one of these IOP-independent risk factors for NTG arising via a novel mechanism.

In the present study, we examined fundus photographs from PA patients to determine if PA is associated with glaucoma-related fundus abnormalities in order to evaluate the possibility that PA may be a candidate responsible for glaucomatous optic neuropathy.

## Methods

### Study participants

This study consisted of a retrospective review of case records. A total 178 patients who were suspected to have primary aldosteronism and had undergone examinations including adrenal venous sampling (AVS) and fundus photographs at Kanazawa University Hospital from 2011 to 2016 were included. Eyes with poor fundus images or cases without a final diagnosis of PA were excluded. As controls, 354 eyes from 177 cases with good quality fundus photos were randomly selected, matching for age and sex, from among 1173 examinees that participated in a public glaucoma screening performed at Yawata Medical Center [18]. The study was part of a community health screening in Komatsu City, Ishikawa, Japan. Participants of the glaucoma screen underwent examinations including noncontact pneumotonometry and nonmydriatic fundus photography [18]. This study was approved by the Medical Ethics Committee of Kanazawa University Hospital (No. 2015–246), and followed the tenets of the Declaration of Helsinki. Informed consent for the study examinations was obtained from each participant.

### Examination

Fundus photos of all PA patients were obtained using a nonmydriatic fundus camera. PA patients also underwent health examinations consisting of anthropometry, blood pressure measurements and blood tests. Pretreatment blood pressure was obtained from their medical records. Adrenal venous sampling tests were conducted to confirm a diagnosis of PA. The control group completed medical history questionnaires to identify the presence of hypertension, diabetes mellitus or heart disease.

### Diagnosis of PA

Diagnosis of primary aldosteronism was based on The Japanese Society of Hypertension guidelines for the management of hypertension (JSH 2014) [4]. All patients in the PA group underwent a blood sample screening to examine plasma aldosterone concentrations (PAC, ng/dL) and plasma renin activity (PRA, ng/mL/h). At least one of the following PA confirmatory tests was also conducted: fludrocortisone suppression test, saline infusion test, oral sodium loading test or captopril challenge test. A diagnosis of PA was confirmed if the aldosterone-to-renin ratio (ARR) exceeded 200, and at least one confirmatory test was positive.

### Evaluation of fundus photos

The color fundus photos and their red-free images for RNFL detection [13, 19] were arranged side-by-side and examined by all members of the study group which included 2 glaucoma specialists (T.H. and K.S.). The fundus photos suspected to show abnormalities were reevaluated and the judgment was finalized by agreement of the two glaucoma specialists.

Glaucomatous optic disc appearance was graded according to the International Society of Geographical and Epidemiological Ophthalmology (ISGEO) criteria and the Tajimi study [10, 20]. Given that no visual field tests were available for the PA patients, eyes that were rated category 2 (advanced structural damage with unproved field loss), i.e. ≥ 0.9 cup-to-disc ratio, ≤ 0.05 of the disc diameter of the rim width in the superior (11–1 hours) or inferior (5–7 hours) sectors, or ≥ 0.3 difference in the vertical cup-to-disc ratio between both eyes, were regarded as “glaucomatous”. Eyes with a cup-to-disc ratio of the optic nerve head of 0.7 or greater but less than 0.9, the rim width at the superior (11–1 hours) or inferior (5–7 hours) sector of 0.1 or less but more than 0.05 of the disc diameter, or a difference in the vertical cup-to-disc ratio between both eyes of 0.2 or greater but less than 0.3, were regarded as “glaucoma suspects”. The glaucoma status of each person was classified based on the more affected eye.

RNFLDs were defined as wedge-shaped darker regions, wider than the major retinal vessels [21]. RNFLDs were classified by their central ends: (1) at the optic disc margin, or (2) adjacent to the retinal vessels, such as blood vessel bifurcations or arteriovenous intersections, without reaching the disc margin. The location of RNFLDs in the upper or lower hemiretinas was also documented. RNFLDs that reached the disc margin where a glaucomatous optic disc appearance was present were regarded as glaucomatous RNFLDs.

### Statistical analysis

The mean values (presented as the mean ± SD) were reported for continuous variables. Differences between the PA group and control group, or PA group with or without RNFLDs were evaluated using a Mann Whitney U test or chi-squared test for each variable.

Mixed-effects logistic regression models using clustered robust standard errors were used to compare the glaucoma–related fundus abnormalities per eye between the PA and control groups accounting for the correlation of fellow eyes in the same patient. Furthermore, factors associated with the presence of RNFLDs were evaluated per hemiretina using mixed-effects logistic regression models with patient-specific and eye-specific random effects. All data were analyzed with statistical software (STATA, version 15.1; StataCorp LP, College Station, Texas, USA).

## Results

In the PA group, 30 cases that did not satisfy the diagnosis of PA and 24 eyes with poor fundus images were excluded; in total, 272 eyes from 137 cases were analyzed. The demographic data are shown in Table 1. In the PA group, the prevalence of hypertension and diabetes mellitus was significantly higher than in the control group (p<0.001, p = 0.01, respectively). The percentage of glaucoma or glaucoma suspects did not differ significantly between the two groups (p = 0.901, 0.713, respectively).

**Table 1:** Comparison of demographics between the PA and control groups.

Factors	PA, n = 137	Control, n = 177	P value
Age (years)	55.3 ± 10.7	56.1 ± 10.1	0.742
Male (%)	73 (53.3)	89 (50.3)	0.598
Hypertension (%)	135 (98.5)	31 (17.7)	<0.001
Diabetes mellitus (%)	26 (19.0)	15 (8.9)	0.01
Heart disease (%)	9 (6.6)	13 (7.6)	0.716
Glaucoma (%)	5 (3.7)	6 (3.4)	0.901
Glaucoma suspects (%)	4 (2.9)	4 (2.3)	0.713

PA = primary aldosteronism.

Chi-squared test or Mann-Whitney U test.

**Fig 1:**
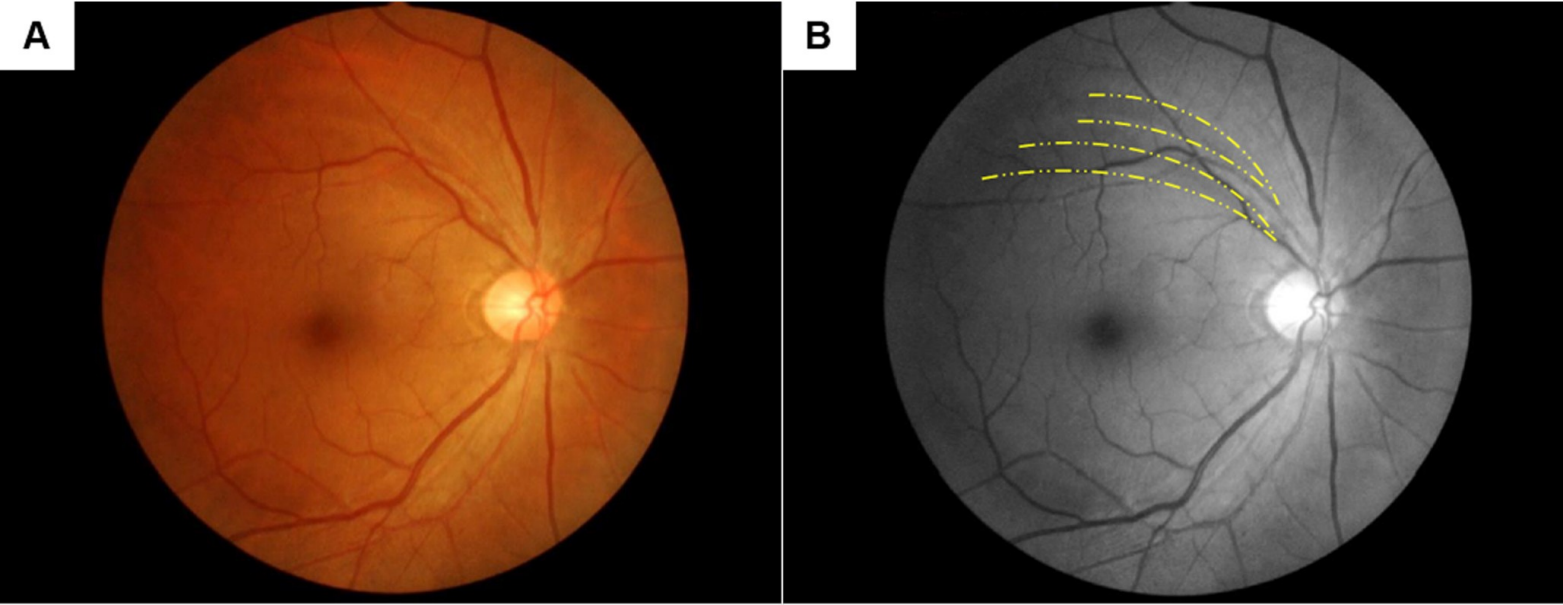
RNFLDs with their central ends around the retinal vessels. Representative fundus photos of non-glaucomatous RNFLDs with their central ends around the retinal vessels. Yellow dotted lines indicate the RNFLDs. This case is from the PA group and shows two RNFLDs with their central ends adjacent to the superior retinal vein and artery. (A) Color photo, (B) Red-free image.

Table 2 shows the differences in glaucoma–related fundus abnormalities per eye between the PA and control groups. Optic disc abnormalities that met the glaucoma criteria were detected at similar rates, 7 eyes in both the PA (2.6%) and control groups (2.0%; p = 0.686). The number of eyes with optic disc abnormalities that met the glaucoma suspect criteria did not differ significantly between the PA (5 eyes, 1.8%) and control (7 eyes, 2.0%) groups (p = 0.900). RNFLDs were detected significantly more often in the PA group than in the control group (p<0.001). In particular, RNFLDs with their central ends around the retinal vessels were much more common in the PA group (26 eyes, 9.6%) than the control group (4 eyes, 1.1%; p<0.001). Fundus photos of a representative case are shown in Fig 1.

**Table 2:** Comparison of glaucoma-related fundus abnormalities between the PA and control groups (per eye).

	PA, n = 272	Control, n = 352	OR (95% CI)	P value
**Optic disc abnormalities (%)**	12 (4.4)	14 (4.0)	1.11 (0.42–2.94)	0.83
Glaucoma* (%)	7 (2.6)	7 (2.0)	1.30 (0.37–4.61)	0.68
Glaucoma suspects* (%)	5 (1.8)	7 (2.0)	0.92 (0.29–2.91)	0.89
**RNFLD (%)**	55 (20.2)	26 (7.4)	3.18 (1.81–5.57)	<0.001
Disc (%)	35 (12.9)	25 (7.1)	1.93 (1.05–3.55)	0.034
Vessel (%)	26 (9.6)	4 (1.1)	9.20 (3.10–27.2)	<0.001
**RNFLD (%) in eyes with optic disc abnormalities**	12 (4.4)	12 (3.4)	1.30 (0.48–3.54)	0.60
Disc (%)	12 (4.4)	12 (3.4)	1.30 (0.48–3.54)	0.60
Vessel (%)	4 (1.5)	1 (0.3)	5.24 (0.52–6.16)	0.16

PA = primary aldosteronism; OR = odds ratio; CI = confidence interval; RNFLD = retinal nerve fiber layer defect; Disc = RNFLDs with their central ends at the disc margin; Vessel = RNFLDs with their central ends adjacent to the retinal vessels.

*Optic disc abnormalities meeting the criteria of glaucoma or glaucoma suspects.

Logistic regression model using clustered robust standard error.

**Fig 2:**
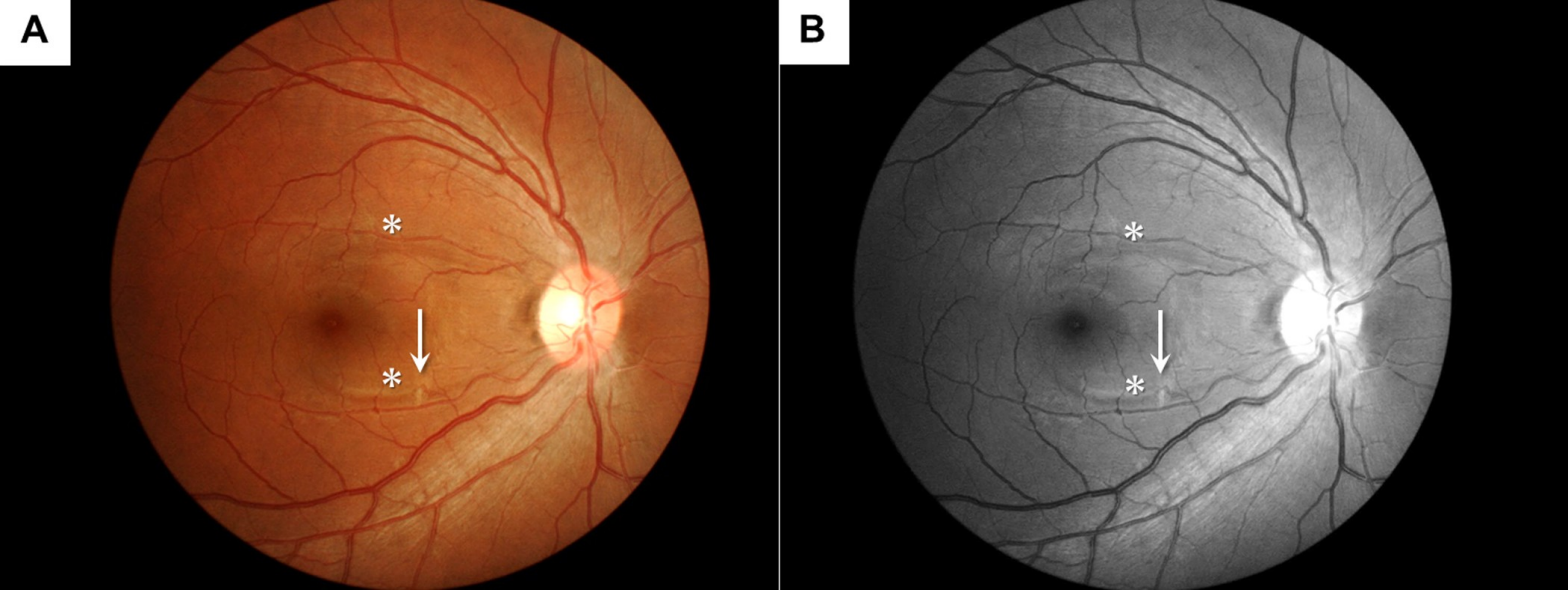
Cotton-wool spot found in the right eye of a PA patient. Fundus photos of the right eye of a 35-year-old man with PA. There were two narrow RNFLDs in the macular region (asterisks). A small cotton-wool spot was observed in the middle of the lower RNFLD (arrow). RNFLDs were judged to be almost touching the margin of the non-glaucomatous optic disc. (A) Color photo, (B) Red-free image.

One PA patient had a fundus abnormality aside from the glaucomatous optic disc changes or RNFLDs that consisted of a small cotton-wool spot (CWS) in the middle of a narrow RNFLD (Fig 2).

After excluding subjects with optic disc abnormalities that met the criteria of glaucoma or glaucoma suspects (17 eyes from the PA group and 20 eyes from the control group), non-glaucomatous RNFLDs were present significantly more often in the PA group than in the control group (Table 3).

**Table 3:** Comparison of RNFLDs between the PA and control groups in subjects without optic disc abnormalities that met the criteria of glaucoma or glaucoma suspects (per eye).

	PA, n = 255	Control, n = 332	OR (95%CI)	P value
**RNFLD (%)**	42 (16.4)	13 (3.39)	4.84 (2.41–9.71)	<0.001
Disc (%)	23 (9.0)	12 (3.6)	2.64 (1.21–5.73)	0.014
Vessel (%)	21 (8.2)	2 (0.6)	14.8 (3.39–64.6)	<0.001

PA = primary aldosteronism; OR = odds ratio; CI = confidence interval; RNFLD = retinal nerve fiber layer defect; Disc = RNFLDs with their central ends at the disc margin; Vessel = RNFLDs with their central ends adjacent to the retinal vessels.

Logistic regression model using clustered robust standard error.

Table 4 shows the factors associated with the RNFLDs with their central ends at the disc margin in the PA group, control group, or in all subjects. PA (p = 0.001), glaucoma or glaucoma suspects (p<0.001), and superior hemiretina (p = 0.037) were independent risk factors for RNFLDs in all subjects. The odds ratio was highest for glaucoma or glaucoma suspects in all 3 analyses. In addition to glaucoma or glaucoma suspects, superior hemiretina was also a significant factor for RNFLDs in the PA group (p = 0.011), but not in the control group.

**Table 4:** Factors associated with the presence of RNFLDs with their central ends at the disc margin (per hemiretina, multivariate analysis).

	PA		Control		All	
	OR (95% CI)	P value	OR (95% CI)	P value	OR (95% CI)	P value
PA	NA		NA		3.52 (1.56–7.92)	0.002
Male	0.66 (0.16–2.69)	0.56	0.37 (0.13–1.10)	0.07	0.52 (0.24–1.15)	0.11
Age	1.03 (0.95–1.10)	0.49	0.98 (0.93–1.04)	0.56	1.00 (0.96–1.04)	0.94
HT	NA		0.42 (0.06–2.82)	0.38	NA	
DM	1.82 (0.32–10.3)	0.50	2.92 (0.63–13.5)	0.17	1.90 (0.71–5.12)	0.20
Superior hemiretina	3.71 (1.36–10.1)	0.011	1.12 (0.44–2.88)	0.81	1.94 (1.04–3.60)	0.037
Glaucoma or GS	750.9 (30.6–18399)	<0.001	41.0 (14.4–116.7)	<0.001	112.5 (26.8–472.3)	<0.001

PA = primary aldosteronism; OR = odds ratio; CI = confidence interval; NA = not applicable; HT = hypertension; DM = diabetes mellitus; GS = glaucoma suspects.

Table 5 shows the factors associated with the presence of RNFLDs with their central ends adjacent to the retinal vessels in all subjects. These RNFLDs were associated with PA (p <0.001) and glaucoma or glaucoma suspects (p = 0.018) in all subjects. The odds ratio for PA was higher than that for glaucoma or glaucoma suspects.

**Table 5:** Factors associated with the presence of RNFLDs with their central ends adjacent to the retinal vessels in all subjects (per hemiretina, multivariate analysis).

Factors	OR (95% CI)	P value
PA	19.9 (4.06–97.8)	<0.001
Male	1.51 (0.50–4.56)	0.47
Age	0.97 (0.92–1.02)	0.25
DM	1.07 (0.24–4.67)	0.93
Superior hemiretina	1.28 (0.57–2.87)	0.54
Glaucoma or GS	9.26 (1.46–58.6)	0.018

PA = primary aldosteronism; OR = odds ratio; CI = confidence interval; DM = diabetes mellitus; GS = glaucoma suspects.

Next, we compared various factors between non-glaucomatous PA patients with and without RNFLDs at the per person level. There were no significant differences between the two groups, but the aldosterone-to-renin ratio tended to be higher in the group with RNFLDs (p = 0.085; Table 6). Furthermore, mixed-effects logistic regression models with patient-specific and eye-specific random effects showed that plasma aldosterone concentrations (ng/dL) and the aldosterone-to-renin ratio were significant risk factors for the presence of RNFLDs when examined per hemiretina (odds ratios were 1.004 and 1.0004, p values were 0.026 and 0.029, respectively). The superior hemiretina was also a significant risk factor for RNFLDs adjusting for plasma aldosterone concentration (odds ratio, 2.42; p = 0.025).

**Table 6:** Comparison of factors between non-glaucomatous PA patients with and without RNFLDs.

Factor	PA without RNFLD	PA with RNFLD	P value
	n = 94	n = 34	
Male (%)	51 (54.3)	17 (50.0)	0.670
Age (years)	54.9 ±10.7	55.2 ± 11.4	0.867
Hypertension (%)	92 (97.9)	34 (100)	0.391
Diabetes mellitus (%)	17 (18.1)	6 (17.7)	0.955
Heart disease (%)	8 (8.51)	1 (2.94)	0.276
Dyslipidemia (%)	30 (31.9)	12 (35.3)	0.719
Cerebrovascular disease (%)	4 (4.3)	4 (11.8)	0.121
SAS (%)	5 (5.32)	2 (5.88)	0.901
Pretreatment SBP (mmHg)	158 ± 19.8*^1^	154 ± 14.8*^2^	0.618
Pretreatment DBP (mmHg)	99.7 ± 12.1*^1^	94.6 ± 8.39*^2^	0.253
BMI (kg/m^2^)	25.2 ± 4.5	24.5 ± 3.7	0.850
SBP (mmHg)	137 ± 17.8	137 ± 20.2	0.740
DBP (mmHg)	84.1 ± 12.3	86.5 ± 13.1	0.419
PAC (ng/dL)	142.5 ± 69.6	193.9 ± 197	0.352
PRA (ng/mL/h)	0.4 ± 0.2	0.32 ± 0.19	0.421
ARR	543.7 ± 473.1	1015 ± 2022	0.085

RNFLD = retinal nerve fiber layer defect; SAS = sleep apnea syndrome; SBP = systolic blood pressure; DBP = diastolic blood pressure; BMI = body mass index; PAC = plasma aldosterone concentration; PRA = plasma renin activity; ARR = aldosterone to renin ratio.

*^1^ n = 41,

*^2^ n = 11.

Chi-squared test or Mann-Whitney U test.

## Discussion

In this study, we investigated the relationship between primary aldosteronism and glaucoma-related fundus abnormalities. The prevalence of glaucoma or glaucoma suspects based on optic disc appearance was 6.6% in PA patients, which did not differ significantly from the control group in our study. However, RNFLD, another glaucoma-related fundus abnormality, was detected at a significantly greater frequency in PA patients, especially for RNFLDs with their central ends located around the retinal vessels.

The possible adverse effects of PA on vision include fundus abnormalities associated with systemic hypertension, such as severe hypertensive retinopathy, retinal vascular occlusion, and non-arteritic ischemic optic neuropathy [6, 7]. In our study, there was only one case with a CWS that could be regarded as grade III hypertensive retinopathy based on the Keith-Wagener-Baker classification [7, 22]. As for other vision-threatening diseases, Van Dijk et al. reported that retinal abnormalities resembling central serous chorioretinopathy were frequently observed in PA patients, and speculated the potential involvement of the mineralocorticoid receptor-mediated pathway in the pathogenesis of the disease [8]. In our present cases, no eyes showed any apparent fundus abnormalities indicative of central serous chorioretinopathy.

In regard to glaucoma, recent reports demonstrated that systemic administration of aldosterone in rat models resulted in progressive RGC loss and glaucomatous optic nerve degeneration without elevated IOP [9]. Moreover, Ono et al. [23] reported that systemic administration of aldosterone leads to significant up- or down-regulation of several genes, and suggested that ocular blood abnormalities or apoptosis may be associated with RGC death. In the present study, the prevalence of glaucoma or glaucoma suspects based on optic disc appearance was similar in PA patients and the control group. Although glaucoma prevalence may be underestimated given that glaucoma diagnoses were made using only the optic disc criteria, the prevalence of glaucoma, when glaucoma suspects were also included, was 5.6% in the control group which is similar to results from population-based glaucoma surveys [10, 11]. Therefore, PA may not be a significant contributor to glaucomatous optic neuropathy. In this study, 101 patients were already taking antihypertensive medications and 11 patients had used PA-related medications, such as eplerenone, since the time of their PA diagnosis. While this may not affect the RNFLDs that have already formed, a previous report showed that the administration of a mineralocorticoid receptor blocker prevented RGC loss [9]. This clearly suggests that oral administration of PA-related medications might have suppressed the optic neuropathy due to PA and the formation of RNFLDs.

Although a localized RNFLD is one of the characteristic changes of glaucomatous optic neuropathy, it does not always indicate glaucoma. RNFLDs can also be found in eyes with optic disc drusen, toxoplasmotic retinochoroidal scars, retinal CWSs, and papilledema [24, 25]. Na et al. [26] reported that the estimated prevalence of RNFLDs in Korea was 4.8%, of which 34.4% was glaucomatous and 65.6% was non-glaucomatous. In that study, RNFLDs were defined as reaching the edge of the disc, and glaucoma was diagnosed according to the modified ISGEO criteria including FDT perimetry and RNFLD. The prevalence of RNFLDs was associated with older age, male sex, glycosylated hemoglobin, disc hemorrhage, and a glaucomatous optic disc. The Beijing Eye Study is a population-based study which reported the 10-year incidence of localized RNFLDs. RNFLD was defined as defects running toward or touching the optic disc border, and glaucoma diagnosis was based solely on the ISGEO optic nerve head criteria, which are similar to those used in our study. The incidence of localized RNFLDs in an adult Chinese population was 4.7% ± 0.2%, and 44% of those cases had glaucoma or were glaucoma suspects. RNFLDs were strongly associated with arterial hypertension and higher prevalence of cerebrovascular infarcts, in addition to glaucoma and diabetic retinopathy [27]. Similarly, RNFLDs with their central ends at the disc margin were found in 14 and 11 eyes of subjects with or without glaucomatous optic disc appearance, respectively, in the control group in our study. Thus, a considerable proportion of RNFLDs is supposed to have non-glaucomatous origins even when their central ends reach the disc margin.

In our study, RNFLDs with their central ends at the disc margin were detected more frequently in the eyes of PA patients than in control eyes. Hayreh et al. reported that rhesus monkeys with chronic arterial hypertension and atherosclerosis developed localized RNFLDs without changes in parapapillary atrophy or the neuroretinal rim [28]. Xu et al. reported that localized RNFLDs running toward or touching the optic disc border were found in 7.5% of patients with arterial hypertension, which was significantly more than in control subjects (1.8%; p <0.001) [29]. Furthermore, localized RNFLDs were associated with the grade of arterial hypertension. Thus, the frequent detection of RNFLDs with their central ends at the disc margin in the PA patients in our study is in agreement with previous studies showing the association between this type of RNFLDs and arterial hypertension. Evaluation of the fundus localization of RNFLDs showed that superior hemiretina was significantly associated with this type of RNFLD only in the PA group and was independent from glaucoma or glaucoma suspects.

Glaucomatous fundus changes typically develop as inferior rim thinning with corresponding superior visual field defects [30], and RNFLDs are often observed in the inferotemporal area [31, 32]. In contrast, non-glaucomatous RNFLDs in the superior hemiretina were reported to have an association with diabetes and hypertension [33, 34]. However, previous reports found that inferior visual field loss, corresponding to superior RNFLDs, is also seen in glaucoma patients with diabetes and ischemic changes in the brain [35, 36]. Kiyota et al. reported that tissue blood flow in the superior to temporal optic nerve head measured by laser speckle flowgraphy was associated with the severity of visual field defects and future progression, and speculated that the superior optic nerve head is vulnerable to hypoperfusion. Non-arteritic anterior ischemic optic neuropathy often involves the inferior visual field defects given that the upper anastomosis appears to be less proficient than the lower. Thus, some subtypes of glaucoma might share this pathophysiology with non-arteritic anterior ischemic optic neuropathy [37]. Kiyota et al. also reported that sleep apnea syndrome was significantly associated with lower blood flow at superior to temporal optic nerve head tissue [37]. Moreover, a previous report suggested a relationship between PA and sleep apnea syndrome [38]. Therefore, PA may have contributed to the emergence of RNFLDs in the superior hemiretina.

In this study, the prevalence of RNFLDs was significantly higher in the PA group. Of note, RNFLDs with their central ends adjacent to the retinal vessels were characteristic because this type of defect was mostly associated with PA. There have been some previous reports of non-glaucomatous RNFLDs not associated with the disc margin. Koh et al. [39] reported a case series of localized RNFLDs similar to our study. In their report, RNFLDs occurred subsequent to retinal CWSs and did not reach the disc margin. It is known that RNFLDs may develop after the formation of CWSs [39–44]. Koh et al. considered that glaucomatous RNFLDs and RNFLDs occurring after CWSs both share orthograde and retrograde axoplasmic flow disruptions in the RGC layer; however, the location of the disruption is in the lamina cribrosa and posterior sclera foramen in glaucoma, and in the terminal arteriole and venule in the case of CWSs [39, 45]. The RNFLDs with their central ends adjacent to the retinal vessels that were identified in our study may also stem from similar pathology to CWSs. Only one eye showed a small CWS in association with an RNFLD in our PA cases (Fig 2). Given the transient nature of CWSs [42], more RNFLDs might have emerged following the appearance of CWSs in PA patients. Consistently, several case reports showed that RNFLDs associated with CWSs may cause visual field defects in the corresponding area [39, 41, 44]. Therefore, once CWSs have disappeared, these eyes may be misdiagnosed as glaucoma. However, unlike RNFLDs in glaucomatous eyes, there are no significant changes in the cup-to-disc ratio, cup volume, nor ratio of rim area to disc area in this type of RNFLDs [40].

Regarding the comparison of RNFLDs between glaucoma-only eyes (i.e. glaucomatous eyes in the control group) and PA eyes, all of 12 glaucoma-only eyes had RNFLDs with their central ends at the disc margin, while only one eye had an RNFLD with its central end adjacent to the retinal vessel. In contrast, two types of RNFLDs were found in a comparable number of PA eyes. However, further studies comparing hypertensive patients with and without PA are needed to clarify whether the RNFLDs with their central ends adjacent to the retinal vessels are specific to PA or not.

Among the PA patients, there were no significant differences in medical history, blood pressure, hypertension history or blood test results between those with and without RNFLDs. However, aldosterone-to-renin ratio tended to be higher in patients with RNFLDs. Furthermore, when examined per hemiretina, plasma aldosterone concentration and aldosterone-to-renin ratio were significant risk factors for the presence of RNFLDs. It has been reported that the frequency of comprehensive complications including chronic renal failure, cardiac diseases and sleep apnea correlate with plasma aldosterone concentrations, suggesting an association with the degree of aldosterone excess [46]. Takasago et al. [47] reported a negative correlation between plasma aldosterone concentration and the number of RGCs after systemic administration of aldosterone in rats. Collectively, aldosterone may be directly related to RNFLDs in addition to aldosterone-induced arterial hypertension.

Several limitations of our study should be noted. First, the diagnosis of glaucoma was based solely on optic disc appearance by fundus photos. However, the criteria of glaucoma diagnosis was based on the standard epidemiological criteria (i.e. ISGEO criteria) [20] in which glaucoma and glaucoma suspects were defined according to the degree of glaucomatous structural abnormalities. RNFLDs may be better detected using optical coherence tomography. Visual field tests were also not included in our study design, which may underestimate the prevalence of glaucoma in PA patients. However, the same criteria for glaucoma diagnosis were applied to the control group, and between-group comparisons were performed to examine the association of PA with glaucoma. Further studies with a prospective design including additional tests such as visual field tests and optical coherence tomography are needed to address the issue. Secondly, the medical history of the control group was based on a self-reported questionnaire. Therefore, hypertensive patients may be underestimated, and PA patients may be present among subjects with hypertension in the control group. Finally, given that 98.5% of PA patients had hypertension in this study, it was impossible to analyze the effect of PA on the results independent of hypertension. Accordingly, it is difficult to conclude that aldosterone directly caused the RNFLDs apart from the effects of aldosterone induced hypertension. However, since hypertension is a very heterogeneous disease, the lone effects of chronic blood pressure elevation might be hard to evaluate even with a large number of patients. Further studies with non-PA patients with hypertension may give us some insight into this issue.

In conclusion, from a clinical perspective, the presence of RNFLDs is one of the key fundus findings for the diagnosis of glaucoma. However, in the present study, patients with PA had a significantly greater prevalence of RNFLDs which were not associated with disc cupping or rim thinning. Furthermore, such non-glaucomatous RNFLDs often presented with their central ends adjacent to the retinal vessels. This particular type of RNFLD may be an important finding indicative of non-glaucomatous causes of RNFLDs, especially in PA. Since plasma aldosterone levels, but not blood pressure, were associated with RNFLDs, further studies are warranted to elucidate the direct influence of aldosterone on RGCs in patients with PA.

## Supporting information

S1 File (XLSX)
